# Fostering Children and Adolescents’ Creative Thinking in Education. Theoretical Model of Drama Pedagogy Training

**DOI:** 10.3389/fpsyg.2018.02611

**Published:** 2019-01-07

**Authors:** Macarena-Paz Celume, Maud Besançon, Franck Zenasni

**Affiliations:** ^1^Laboratoire Adaptations Travail-Individu, Ecole Cognition, Comportements et Conduites Humaines, Université Sorbonne Paris Cité, Université Paris Descartes, Boulogne-Billancourt, France; ^2^Center for Interdisciplinary Research, Département Frontiéres du Vivant et de l’Apprendre, IIFR, Université Sorbonne Paris Cité, Université Paris Descartes, Paris, France; ^3^Laboratoire de Psychologie: Cognition, Comportement, Communication (LP3C, EA 1285), Université de Rennes 2, Rennes, France

**Keywords:** drama pedagogy, creativity, drama, education, children

## Abstract

Drama Pedagogy Training (DPT), as other drama-based pedagogies, has been related to several outcomes, including creativity enhancement. This enhancement is commonly proven through the measurement of different creative processes. In our review we systematize characteristics, activities and techniques of DPT that are assumed to be related to creativity in order to have a more comprehensive framework to identify the specific DPT elements that are involved in the enhancement of some of the creative processes of children and adolescents. To this end, we identified five creative processes in experimental studies using DPT: divergent thinking, fantasy and imagination, associative thinking, symbolization, and problem solving. These processes were cross referenced with DPT characteristics, activities, and techniques that were argued to be related to creativity enhancement. Our review will propose a model with two main categories and six elements as follows: (1) *technical drama phases* which emphasizes the role of narrative and embodiment through (a) corporal and vocal training and (b) main drama techniques (e.g., storytelling and improvisation and role-play), and (2) *psycho-pedagogical framework* which emphasizes the role of a dialogic space through (c) playfulness and a (d) collaborative, safe space. We also identified (e) feedback as an important element of DPT which belongs to both drama technical phases and psycho-pedagogical framework categories. Along with the model, we explain the creative outcomes associated to each of these elements as a means to attire the attention to drama-based pedagogies for the development of creativity in the educational setting.

## Introduction

Creativity can be identified in terms of creative thinking through the evaluation of divergent thinking and creative problem solving abilities. These abilities can be enhanced through creativity trainings (Scott et al., 2004), particularly through embodied creativity trainings (Byrge and Tang, 2015) such as drama-based trainings (e.g., Fischer, 1989; Garaigordobil, 2003; Hui and Lau, 2006; Yasar and Aral, 2012; for a review of drama-based trainings: Lee et al., 2015).

A recent meta-analysis conducted by Lee et al. (2015) showed that drama-based trainings (originally presented as Drama-Based Pedagogies) had significant effects on children’s creative thinking. Moreover, throughout of the scope of the cited meta-analysis, we found a vast universe of training and pedagogies that are assumed to have an impact on children’s creative thinking (e.g., Theater of the oppressed, Boal, 1989; Socio-drama, Moreno, 1943, Drama Pedagogy, García-Huidobro, 1996). Drama Pedagogy Training (DPT), is a particular kind of training issued from Drama Pedagogy García-Huidobro’s (1996) that presumes to enhance creative thinking.

The aim of this review is to (a) describe the characteristics of DPT, (b) analyze the specific elements of DPT that should favor creative thinking in children and adolescents, and (c) highlight any specific aspects of creative thinking that are enhanced through these elements of DPT.

For that purpose, we will first describe the conceptual implications of the concept DPT and how it can be found in literature under different names. Secondly, we will briefly review empirical evidence on how DPT or activities issued from DPT enhanced creative thinking. Finally, we will categorize the elements of DPT that might be enhancing creative thinking according to the current literature and organize them in a model for better understanding. This conceptual model will be presented in two main categories (1) Drama Technical Phases which focuses on the practical activities of DPT including (a) corporal expression training and (b) main drama techniques, such as storytelling or improvisation and role-play, and (2) Psycho-Pedagogical Framework which focuses on the characteristics of a DPT session including (c) playfulness, (d) collaborative and safe environment, and (e) feedback.

## Drama Pedagogy Training (DPT)

In order to understand the concept of DPT, we first need to define Drama Pedagogy. Drama Pedagogy is an active pedagogy based on drama games and techniques. According to García-Huidobro (1996, 2004), it can be divided into four tendencies, namely (a) neoclassical, (b) liberal progressivism, (c) radical, and (d) critical socialism. These tendencies can be inserted in three different areas or dimensions of work, namely (a) inside the educational setting, (b) outside the educational setting, and (c) as a therapeutic dimension. Several combinations of these tendencies and dimensions map onto the concepts reviewed by Lee et al. (2015), even if Drama Pedagogy itself was out of the scope of that study. Thus, DPT can be described as the kind of training that follows the liberal progressivism tendency from Drama Pedagogy inside and outside of the educational setting.

The liberal progressivism tendency of Drama Pedagogy is focused on the experiences that participants can develop through playing drama games rather than preparing a show for an audience (Freeman et al., 2003). In other words, the aim is to contribute to the development of different competencies, such as creativity, by focusing on the process of learning over an artistic result (Heathcote et al., 1991; García-Huidobro, 1996; Woodson, 1999; Libman, 2001; Karakelle, 2009). When Drama Pedagogy is inserted inside the educational setting through the perspective of the liberal progressivism tendency, it helps the development of integral and creative children^1^ (Hui and Lau, 2006; Karakelle, 2009; Cremin and Macdonald, 2013).

In summary, DPT can be considered any training or workshop created through drama activities and techniques (such as pretend play, improvisation, role-play, etc.) that follows the characteristics of the liberal progressivism tendency from Drama Pedagogy. Thus, DPT can be understood as a drama-based training that works with the internal world of the participants (García-Huidobro, 1996) in order to develop creative and socio-emotional competencies, and is focused on the process of learning experiences over artistic or academic results.

For this review, the concept DPT will be used to gather all the training procedures that apply to the characteristics described above, as well as the concepts presented by Freeman et al. (2003) (e.g., child-drama, play-making, child-play, or educational-drama), and the more complex process-oriented methodologies such as socio-drama (Moreno, 1943, 1947; Pecaski, 2012), Creative Drama (Rosenberg, 1981; McCaslin, 1996) or Applied Theatre (Holland, 2009). Overall, this would include all drama-based trainings or pedagogies in which the focus is on the final artistic result or any academic result (e.g., Walker et al., 2011) will not qualify as DPT.

### DPT Improves Creativity: Empirical Evidences

Moreno (1943); Slade (1967), and Boal (1989) can be considered pioneers in DPT as they started the use of dramatic art through representational games in their work with people without searching for any educational nor artistic outcome. Years later, their legacy is still a matter of interest in social sciences, particularly when relating DPT to creativity. In the following section, we will describe the findings of 10 studies relating to creative thinking and DPT in children and adolescents. The analyzed studies correspond to the work of drama-based trainings or pedagogies that match the characteristics of DPT as described above. Some studies have been excluded as the objectives of the training were academic or artistic, or because the particular population presented problems that could lead to a bias, as in the case of children with special needs.

Of all the studies analyzed, only one, conducted in Amato et al. (1973) on 298 school-age children could not prove a significant impact of DPT (originally described by the authors under the name of Creative Dramatics) on children’s creative thinking. The researchers looked for significant differences between DPT and other trainings by measuring children’s creative thinking through the Torrance Tests of Creative Thinking (TTCT) (Torrance, 1990). According to the authors, storytelling training (which is considered in this papers as an element of DPT) had a higher influence on creative thinking than DPT. Nevertheless, a more detailed methodology from the authors was needed in order to confirm their results. We are not aware of the activities carried on in the different training techniques, so we cannot establish a clear difference between the two mentioned trainings and between the control group and the DPT. The article did not provide the means of each group, so no effect size could be calculated.

Ten years later, Clements et al. (1982) conducted a study on 37 adolescents between 13 and 17 years old in order to evaluate the effects of a DPT program on creativity. Adolescents’ creative thinking was measured through the TTCT Demonstrator (Torrance, 1979) and a seven point rating scale was created by two judges in order to measure talent and experience in drama. Results showed significant correlations between TTCT scores and drama experience (*r* = 0.55, *p* < 0.001) and TTCT scores and drama talent (*r* = 0.46, *p* < 0.001) suggesting that children with more experience or talent in drama score better in creative thinking tasks. Results provided suggest medium to large effect sizes.

Similarly, Berretta and Privette (1990) studied 184 school-age children in a fourth grade class, looking for a difference between flexible art, drama, and playground activities versus structured art, drama and playground activities. Findings showed that even though there were no significant effects in the type of activity, flexible activities produced significant differences in creative thinking scores on the TTCT (Torrance, 1990) tests versus the structured ones (*F*_(1,178)_ = 4.08, *p* < 0.05). The effect size of flexible drama and structured drama was analyzed by calculating Cohen’s d based on the mean scores provided by the original article, showing a small effect size (*D* = 0.26). Flexible drama activities, are more related to the concept of playfulness in DPT, where the child is free to construct play following the basic rules of a drama game (McCaslin, 1968).

Authors such as Garaigordobil (1995) have shown that creative drama games, among other cooperative games, enhanced creative thinking (particularly divergent thinking) in a sample of 154 children aged 9–10 years old. Children were assessed using three tasks of the battery of tests Guilford’s (1951). Results showed significant differences in the analyses of variance and between the two groups for all three subtests. We calculated Cohen’s d effect sizes on fluidity scores for the three subtests applied, finding large effect sizes for inusual tests (*D* = 1.4) and circle test (*D* = 0.98), and a huge effect size for the consequence test (*D* = 2.8). In 2003 she created different programs (Programa Juego) for children aged from 8 to 12 years old in order to develop creativity and other social competencies such as altruistic behavior through creative drama games and other cooperative games.

A Chinese team, leadered by Hui and Lau (2006) gathered 126 children attending first and fourth grade in order to measure the impact of DPT (originally described under the name of creative drama) on creative thinking. Children participated in an 1-h-a-week DPT, during 16 weeks. They were tested on creative thinking through the Wallach-Kogan Creativity Test (WKCT; Wallach and Kogan, 1965) and the Test of Creative Thinking Drawing Production (TCT-DP; Urban and Jellen, 1996), while also designing a Story Telling Test (STT) to indirectly measure creativity, finding that participants who attended the training generated more creative responses and drawings than the control group. No standard deviation scores were provided for the WKCT and the TCT-DP tests, so no effect size could be calculated. For the STT, Cohen’s d analysis showed small to medium effect size (*D* = 0.37) on the creativity item.

In a study conducted by Yeh and Li (2008), 116 children aged 4 to 6 years old, participated into a study to see the impact that drama might have on children’s creativity. He created the Preschoolers’ Creativity Test in order to measure usefulness and novelty indices, finding that the drama training through the different participants age had significant effects on children’s creativity with large effect sizes (*F*_(2,112)_ = 42.27, *p* < 0.001, η^2^ = 0.43).

Mullineaux and Dilalla (2009) conducted a longitudinal study on 120 children engaged in a 20 min free pretend-play (one fundamental element of DPT). They used a realistic role-play rating to measure pretend-play at age 5, and WKCT Uses-Tasks (Wallach and Kogan, 1965) plus TCT-DP (Urban and Jellen, 1996) to measure creative thinking at age 10–15. Results showed that Realistic-Role-play at age 5 was significantly correlated to WKCT (*r* = 0.18, *p* < 0.05) and TCT-DP (*r* = 0.22, *p* < 0.05) scores at follow-up, suggesting that pretend play is predictor of later creative thinking. Results of correlations suggests medium effect sizes.

Lin (2010) studied 67 children aged 11–12 years old engaged in a 10 week intervention two-sessions-a-week DPT. He tested how a drama intervention could enhance possibility thinking, as a facet of creative thinking, through diaries, response sheets and group interviews. Findings showed that children considered the drama training as a useful tool for fostering their creativity. Results from this study are qualitative, so no effect sizes could be calculated.

Garaigordobil and Berrueco (2011) in Spain, tested a play based training including drama activities on 86 participants aged 5 and 6. Participants engaged in a 75 min long training sessions each week throughout the academic year. The researchers measured creativity through TTCT and the Escala de Personalidad Creadora (EPC; Garaigordobil and Pérez, 2005) finding that the training significantly increased creative personality traits, as well as verbal (e.g., fluency scores: *F*_(1,84)_ = 39.99) and graphic (e.g., fluency scores: *F*_(1,84)_ = 15.31) creative thinking. They also concluded that was the positive atmosphere what enhanced children’s creativity. Cohen’s d were calculated for fluency scores in the TTCT test, showing large to huge effect sizes for verbal (*D* = 1.89) and graphic (*D* = 0.97) tasks.

One year later, Yasar and Aral studied 80 children aged 6 years-old who participated engaged in a twice a week in a drama education workshop over the course of 12 weeks. They were tested through TCT-DP forms A and B showing significant differences between the experimental and the control group (*t* = 16.1, *p* < 0.000). Besides, we calculated Cohen’s d presenting a huge effect size (*D* = 3.11).

Finally, in, Mottweiler and Taylor (2014) studied the effect of elaborated role play on 75 children aged 4 to 5 years-old. Results showed that children who participated in elaborated role play had higher creativity scores with small to medium effect sizes on narrative (*F*_(l,68)_ = 6.31, *p* < 0.039, η^2^ = 0.06) and drawing (*F*_(l,49)_ = 3.08, *p* = 0.09, η^2^ = 0.06) creativity tasks.

A recent study, made by Hainselin et al. (2018), showed how improvisational theater helped enhance divergent thinking on 35 adolescents, measured through Alternative Uses Task (AUT; based on Guilford, 1967). Nevertheless, even though improvisation is part of DPT, in their study improvisation is considered as a the methodology used for adult actors or adult population, while in our study improvisation is considered as improvisational games within a particular framework (we will detail the framework below). In this line, the study of Hainselin et al. (2018) and other similar works using improvisational theater, should be considered with caution, and only as partial evidence for our study.

Effect sizes are varied for our simple. No mean for effect sizes was calculated, as some effect sizes were calculated via Cohen’s d and others were provided by ANOVA analyses. Three out of 11 studies, presented problems to find effect sizes or to calculate Cohen’s d. Eight studies presented sufficient data, and thus effect sizes were calculated. Two out of eight studies presented small or small to medium effect sizes, two out of eight presented medium or medium to large effect sizes and four out of eight presented large, large to huge, or huge effect sizes.

Although the studies described above make a consensus that DPT have an influence on creative thinking, none of these works explained nor differentiated which are the specific elements or characteristics of DPT that are involved in the enhancement of the different facets of creative thinking. Consequently, below we propose a model that aims to explain and categorize the different elements of DPT that are involved in the development of creative thinking.

## DPT Model for Creative Thinking

According to the literature reviewed above, successful creativity trainings are based on different DPT elements (e.g., Baker, 1996; Freeman et al., 2003). Following this review, we propose to categorize the elements identified into two types of main categories (1) Drama Technical Phases and (2) Psycho-Pedagogical Framework.

The Drama Technical Phases category describes the practical drama-based activities carried out during the DPT training and emphasizes the role of narrative and embodiment through the following elements: (a) corporal expression training (e.g., Byrge and Tang, 2015) and (b) main drama techniques, such as storytelling (Laidlaw, 2000) or improvisation and role-play (e.g., Moreno, 1943; Johnstone, 2012).

The Psycho-Pedagogical Framework category describes the characteristics of a DPT session and emphasizes the role of a dialogic space (including some of the aspects of narrative) through the following elements: (c) playfulness and a (d) collaborative and safe environment.

We also identified (e) feedback as an element of DPT which will be taken as an intersection between drama technical phases and psycho-pedagogical framework categories.

**FIGURE 1:**
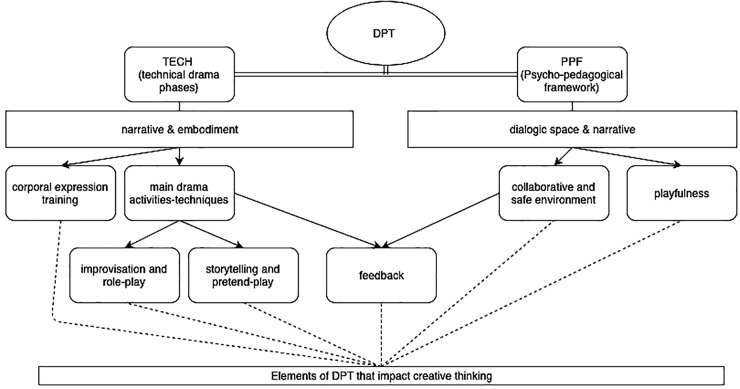
Model of the drama pedagogy training elements that impact creative thinking.

In order to illustrate these findings, please see the proposed model in Figure 1.

In the following section we will present each of the previously identified DPT elements discussing their influence on the creativity outcomes and creativity related processes proposed by the current literature.

### The Drama Technical Phases Category

#### Corporal Expression Training

Corporal expression training can be understood as the physical training that happens at the beginning of a drama pedagogy session. For instance, participants are asked to move around the space and to explore the different movements they can use to move from one point of the room to another, changing speeds and quality of movements at each time.

The use of the body in support of expression is commonly related to dance. Nevertheless, one fundamental part of dramatic art is the ability to express emotions and thoughts through the body (Snow, 2012). Corporal expression training enables the whole body for being capable to adapt to any situation that might arise (Barba, 1979). Our bodies are the first mediators between the self and the environment because all of our words and emotions go firstly through our bodies before being coded by our brains (Aden, 2014). Consequently, embodiment as a relationship between feelings, ideas, thoughts, behaviors and kinesthetic experiences (Barsalou, 1999), would be the first step to any emotional or cognitive process including creativity, which would develop as a consequence of a particular interaction between the body and its environment. For Martínez and Díaz (2006) motor creativity is the ability to produce fluid, different and novel answers in order to solve a motor function or expression. They explain that when motor tasks are more ambiguous, several cognitive resources work together for the resolution of the task. According to some authors, we can talk of creative task resolution when productions are both novel and adapted to a context (Runco and Jaeger, 2012; Lubart et al., 2015). Further to this, corporal-expression in DPT could also be considered creative if the use of body is original and adapted to the context in which it’s being performed. Thus, a movement could be considered *original* if participants express themselves in an uncommon way, while *adaptation* would be related to the ability of the performer to express him/herself accordingly in the space in an *organic/authentic* way. In other words, being able to “fill the empty space” with his/her body in order to clearly transmit ideas or emotions (Brook and Gil Novales, 1997).

Goodwin (1992) suggested that an *authentic movement* creativity is a response that comes from the consciousness of the unity of *body (or experience) with mind (or interpretation)*. When DPTs involve communication between mind and body participants are able to find solutions to conflicts that they would be unable to if this connection was not established (Goff and Torrance, 1991). Creativity is a flux of movement from the unconscious to the conscious body (Halprin, 2014), achieved as a result of the embodiment process. Thus, through corporal expression, we have access to personal information that enables us to create something original and authentic, based on the interrelation of the unconscious and the conscious body. Embodiment is a powerful tool with which to develop creativity (Byrge and Tang, 2015) as the act of experiencing being someone else leads participants to take risks and explore the unknown (Yaniv, 2012), influencing creativity. Moreover, if we consider creativity from perspective Kharkhurin’s (2014), authenticity is the ability to express the inner self by giving meaning to the creative act, increasing its’ aesthetic value. Authentic movement is a creative process in which ideas are transformed into a creative act by showing the performer’s inner self.

From another perspective, Sofia (2016) proposed that the motor functions of the brain and the motor system are the basis of human thinking, supporting the theory of mirror neurons (Rizzolatti et al., 1996). This demonstrates that creative processes in performers does not result in a creative a piece of art, but explains creativity as an effective relationship with the audience through an adjustment of the body–mind relationship. From this perspective, creativity is achieved through a process of embodiment that results in an empathetic connection between the performer and the audience. Both performer and spectator create a novel and adapted product, transforming reality.

In summary, corporal expression in DPT directly affects creativity through enhancing problem solving abilities, but it also works by developing risk taking and tolerance of the unknown that is achieved through a process of embodiment.

##### Main drama techniques: Storytelling and Pretend-Play

Storytelling and Pretend-Play are both narrative activities (Nicolopoulou, 1997). Pretend play, also called dramatic play (Barbour, 2016) is the ability to narrate a story (Laidlaw, 2000) through enacting narrative scenarios while storytelling uses a discursive exposition of them (Nicolopoulou, 2015). To clarify, storytelling is a way of organizing and telling a story while pretend-play is the enactment of a story. In DPT storytelling is the moment when participants create their stories and organize them in a way that makes sense to them. Pretend-play in this case, is the way children pretend to use objects that do not exist, and generate actions or sounds, but do not necessarily represent a character (this would be defined as role-play). For example, some children are asked to tell a story (storytelling) while other children create the sounds or represent the activities told within the story (pretend-play).

Several authors associate pretend-play with children’s creativity (e.g., Dansky and Silverman, 1973; Dansky, 1980; Fein, 1981; Singer and Singer, 1990; Urban, 1991; Moore and Russ, 2008; Mullineaux and Dilalla, 2009). Goff and Torrance (1991) have shown that enactment in DPT helped participants to visualize different solutions to a problem fostering their creative, divergent thinking. Similarly, Rosen (1974) and Vandenberg (1980) concluded that through pretend-play children learn about the world enabling them to engage in problem solving creatively. Moreover, Hoffmann and Russ (2012) demonstrated a relationship between storytelling and play, showing that children with greater imagination during play and better organization in their stories (storytelling) were later superior in divergent thinking. They also explain that some cognitive processes related to creativity in pretend-play are divergent thinking and storytelling, highlighting the importance of symbolism and FI abilities. Russ (2004) states that pretend-play allows participants to think of affects and how to express them, enabling children to enhance their ability to connect memories and associations that might help creative problem solving.

Conversely, for Henry (2000) transformation is aided through the creation of metaphors where feeling and imagination interact in order to transmute reality and change the perspective of a situation. Joronen et al. (2011), found that ideas derived within storytelling increased empathy and perspective, thus impacting creativity. Therefore, in DPT the different points of view and imaginations of participants can change the storytelling of an enacted situation, also enhancing perspective taking and transformation. Mellou (1994) states that transforming the present is a cognitive process related to imagination, and that children’s ability to transform objects favors creativity because children are focused beyond the obvious, enabling them to make new associations. Holland (2009) discovered that when children retold their story from different viewpoints to different participants, this helped them to consider different perspectives and create additional, different content. Dramatizing stories directly affects children’s motivation and allows them to think in a more sophisticated way (Wright et al., 2008), helping the development of critical thinking and ideation, developing imagination, and divergent thinking.

In summary, during pretend-play creativity is enhanced through the act of performing narrative situations (*enactment*), enabling the development of perspective, divergent thinking, and problem solving. Storytelling, related to organization in play, helps the development of divergent thinking and imagination, while transforming reality helps develop perspective and empathy.

##### Main drama techniques: Improvisation and Role-Play

Role-play is a specific kind of pretend-play where a person pretends to be someone else in order to portray a character, while improvisation is related to spontaneous acting. In DPT both activities are used in the form of collective games. On the contrary, in adults’ or actors’ improvisational theater participants are pushed to showcase. For example, in collective improvisation games, the facilitator may ask participants to create, in a limited amount of time (improvisation), a collective short scene portraying (role-play) someone who is experiencing a particular emotion.

Goff and Torrance (1991) explain that role-play has been used to find solutions to a wide variety of problems and dilemmas, as we can see in Moreno (1943) who explained that role-playing favors participants’ capacity to analyze everyday issues. Verriour (1989) describes DPT as a tool based principally on improvisation which develops risk taking, decision making, empathy, and perspective. Role-playing sees participants playing “as-if” they are in the shoes of another. According to McCaslin (1968) playing “as-if” increases mental flexibility, helping individuals to solve challenges or examine issues beyond the obvious, while Dansky (1980) claims that when children engage in “as-if” attitudes it enables them to improve their creative performances, particularly associative fluency. Moreover, Mellou (1995) highlights the work of Sutton-Smith (1967) and Bruner (1972) showing how “as-if” play is directly related to flexibility, inferring that both authors agree that role-play in DPT is a contributor of creative development. This capacity to analyze daily issues by playing “as-if” could help develop problem solving skills, and relates to work on perspective-thinking and empathy, as is proposed by San (2002), Laidlaw (2000); Pecaski (2012) or cited in Erdogan (2013). The latter established that role-playing and improvisation helps participants to reorganize their cognitive patterns when reviewing experiences, ideas or behaviors within a group. A study by Goldstein and Winner (2012) showed that drama training enhanced empathy through taking in the perspective of different situations that are represented during DPT sessions, by testing a DPT group against music or visual arts. Empathy has been proved to be related to creativity, as creative problem solving depends on the ability to adapt to situations within the environment, and the ability to empathize with situations (Carlozzi et al., 1995).

Hui and Lau (2006) established that role-playing is fundamental in developing creativity, showing that participants in a role-play based DPT carried out greater boundary breaking and elaboration, but also gave more creative responses in standardized tests (WKCT and TCT-DP) than non-participants. Similarly, Burton and Dalby (2012) showed that role-playing radically increased motivation and idea generation, while Karwowski and Soszynski (2008) showed that role-play training increased divergent thinking through fluency scores of their participants.

Furthermore, in a recent study, Sowden et al. (2015) analyzed how dance and drama improvisation activities can enhance divergent thinking in a sample of 27 children aged 7 to 11. For the first experiment, they worked with improvised dance, and in the second with improvised drama. For the improvised drama intervention, children were measured through the figural activity 1 of the TTCT (Torrance, 1974) and it was found that children who took part in the improvisation group showed better divergent thinking after the intervention compared to those in the control group.

In summary, DPT can improve problem solving and divergent thinking abilities in children and adolescents, favored by mental processes that are developed thanks to improvisation and role-play activities, such as boundary breaking, empathy, perspective taking, risk taking and decision making.

### The Psycho-Pedagogical Framework Category

#### Playfulness

Drama Pedagogy Trainings are always based on play and improvisation (Yeh and Li, 2008). Since Piaget (1952), authors have had confidence in and proved the importance of play for creativity (e.g., Kneller, 1965; Dansky and Silverman, 1973; Lieberman, 1977; Sutton-Smith, 1979; Christie and Johnsen, 1983; Berretta and Privette, 1990; Mellou, 1995; Garaigordobil and Berrueco, 2011; Hoffmann and Russ, 2012) with only a few studies failing to replicate these effects (e.g., Smith and Whitney, 1987).

Contemporary studies show an increase in children’s divergent thinking and associative thinking due to play: Garaigordobil and Berrueco (2011) conducted training throughout the academic year with children aged 5 to 6, demonstrating that there were significant differences in scores (*F*_(1,84)_ = 3.69, *p* < 0.001) on TTCT and Behaviors and Traits of Creative Personality (Garaigordobil, 2006) between play and control groups. Hoffmann and Russ (2012) corroborate these results, concluding that transformation and insight abilities in play are part of creative skills. They worked in a four-session play intervention with children aged 5 to 10, measuring play through the APS and WKCT-Uses-task. The results showed a positive correlation between divergent thinking and organization in play (fluency *r* = 0.32, *p* < 0.05; flexibility *r* = 0.28, *p* < 0.05), and imagination in play (fluency *r* = 0.38, *p* < 0.05; flexibility *r* = 0.36, *p* < 0.01).

The results described above are in part explained because when playing dramatic games, the child experiments with freedom of expression (Jindal-Snape et al., 2011) and exploration and liberation of the self (Winnicott, 1971) through play, which could help the enhancement of associative thinking (Russ, 2004) and divergent thinking (Russ and Grossman-McKee, 1990).

#### Collaborative and Safe Space

Being creative is a participant’s decision (Sternberg, 2002), as creativity is affected by the psychological state of participants and their environment (Hohmann and Weikart, 2002). Even though DPT encourages participants to take risks and be creative (Karakelle, 2009), several factors in the creativity environment and space can impact children’s mood allowing them to be more or less creative (Morrongiello et al., 2015; Celume et al., 2017). For example, a child’s confidence is fundamental in developing a space for free expression (Holland, 2009) which according to Vass et al. (2014) forms part of a co-constructive “interthinking” (Mercer, 2000) in *a* dialogic and free-of-risks space. This free-of-risk play that DPT space offers enables children to increase their willingness to explore other creative dimensions, due to an enhancement of self-confidence and a lack of fear of being in front of others (Farris and Parke, 1993). Furthermore, the act of observing other participants’ performances can impact another participant’s awareness of the differing points of view of a situation as well as different solutions to an issue (Karakelle, 2009), thus developing perspective and problem solving abilities.

In a study conducted in Erdogan (2013) showed that children were able to express themselves as a result of the flexible, free learning setting that their teachers had implemented from DPT. A dynamic environment can enhance children’s free expression allowing them to be more creative. Jindal-Snape et al. (2011) claim that children’s solution finding through a safe environment is not new to DPT as it gives them freedom of expression to find solutions to problems, in a space that permits mistakes. In another study, Garaigordobil and Berrueco (2011) mentioned that one element that fostered children’s creativity was the positive climate generated in the classroom. Similarly, as mentioned in the pretend play section, having a space in which to experience and express positive effects is important for creativity, and playing collaborative games (like those played in DPT) enhances positive feelings helping construct a positive ambiance that leads children to develop imagination and fantasy (Hoffmann and Russ, 2012). For Vygotsky (1967) children’s imagination is developed within child’s play as a response to a collaboration between social interactions, allowing learning to occur. The interactions that accompany this process of learning can be observed in the dialogic space of DPT as *intersubjectivities* (Rogoff, 1990), by describing the process of learning that occurs when learners work collaboratively in order to solve a problem.

In summary, a safe space that permits dialogic interactions to occur will favor creativity through the development of imagination, fantasy, understanding and problem solving abilities as well as by enhancing children’s positive mood allowing participants to be more creative.

#### Feedback

Feedback occurs at the end of each session. Tam (2016) claims that creative and multiple meaning in DPT might not occur when children are engaged in play, but when they are *back into the real world*. Capron-Pruozzo (2014) explained that if children receive positive feedback regarding their creative personality, their creative self-efficacy will be enhanced. She also claims that if children feel they have the means to face different situations, to take risks, to find solutions and persevere, they will be more engaged in dramatic activities, and vice versa. If participants are motivated they will be open to trying new methods and experience, thus impacting creativity.

Pecaski (2012) claims that all sessions should conclude with an oral discussion to enable children to talk about their personal actions, responses, and feelings on the session. Feedback allows children to think critically about how they acted during the session, and put into perspective how they responded to the presented situation or problem, allowing them to rethink about various appropriate solutions to the original problem. Subsequently, feedback enables children to confront problems presented during the session through dramatic games, motivating them to find novel solutions, thus impacting both perception and problem solving abilities.

## Discussion and Conclusion

The spotting of a structure of DPT, helps to understand and clarify past findings on educational drama studies and to design and conduct future research in order to measure the impact of DPT and its particular elements on children’s and adolescents’ creative thinking.

Reviews of studies show how different elements of DPT can be related to either creative outcomes or the related processes of the creative thinking. Some studies clearly explain particular creative outcomes, while others present creativity as a whole. Table 1 presented below intends to summarize the reviewed works in order to present a clearer idea of the relationship between the elements of DPT and the different creative outcomes and creative-related processes.

**Table 1:** Elements of DPT and the corresponding creative-related processes and creativity outcomes.

DPT element	Creative-related process (if any)	Creativity outcome referred	Reference
Corporal expression and training	Adaptation to context		Barba, 1979
	Task resolution	Problem solving	Martínez and Díaz, 2006^∗^
		Problem solving	Goff and Torrance, 1991
	Risk Taking + Tolerance of the Unknown		Yaniv, 2012
	Empathy (audience-performer)		Sofia, 2016
Storytelling and Pretend-play	Problem solving	Divergent thinking	Goff and Torrance, 1991
		Problem solving	Rosen, 1974; Vandenberg, 1980
	Fantasy and imagination, symbolism	Divergent thinking	Hoffmann and Russ, 2012
	Think and express affects; associative thinking	Problem solving	Russ, 2004
	Transformation, imagination		Henry, 2000
	Empathy; perspective taking		Joronen et al., 2011
	Transformation, imagination	Associative thinking	Mellou, 1994
	Perspective taking		Holland, 2009
	Critical thinking; ideation	Imagination, divergent thinking	Wright et al., 2008
Improvisation and role-play	Boundary breaking; elaboration; fluency		Hui and Lau, 2006
	Motivation; idea generation		Dalby and Burton, 2013
	Fluency	Divergent thinking	Karwowski and Soszynski, 2008
		Problem solving	Goff and Torrance, 1991
	Risk taking; decision making; empathy; perspective taking		Verriour, 1989
	Flexibility	Problem solving	McCaslin, 1968
	Associative fluency		Dansky, 1980
	Flexibility		Sutton-Smith, 1967; Bruner, 1972 (in Mellou, 1995)
	Perspective taking; empathy	Problem solving	Laidlaw, 2000; Pecaski, 2012
	Empathy; perspective taking		Goldstein and Winner, 2012
Playfulness	Fantasy, imagination	Divergent thinking	Russ, 2003
	Flexibility (activities)	Divergent thinking (originality)	Berretta and Privette, 1990
		Divergent thinking	Garaigordobil and Berrueco, 2011
	Transformation, insight	Divergent thinking	Hoffmann and Russ, 2012
		Associative thinking; symbolization	Russ, 2004
		Divergent thinking	Russ and Grossman-McKee, 1990
Collaborative and safe space	Risk taking		Karakelle, 2009
	Solution finding		Jindal-Snape et al., 2011
	Imagination		Vygotsky, 1967^∗^
Feedback	Creative self-efficacy		Capron-Pruozzo, 2014
	Critical thinking; perspective taking		Pecaski, 2012


*^∗^Some studies are based on theoretical approaches and not DPT empirical studies; They correspond to the specificity of the use of the element in literature (e.g., Martínez and Díaz, 2006 in Corporal Expression Training; and Vygotsky, 1967 in Collaborative and Safe Space).*

According to literature reviewed, the majority of creative outcomes measured correspond to Divergent Thinking or are related to Problem Solving, and these can mostly be found within the DPT elements of Storytelling and Pretend-play, and Playfulness. Conversely, the DPT element of Improvisation and Role-Play present more creative related processes, such as risk taking or perspective taking rather than specific creative outcomes. Even though we categorized the elements of DPT in order to link them with creative outcomes, some authors (Goff and Torrance, 1991; Hoffmann and Russ, 2012) relate their results to two or more elements of DPT. In the case of Hoffmann and Russ (2012) they make links between creativity and Pretend-play but also Playfulness, which might imply a connection between the proposed elements. Goff and Torrance (1991) observed that creativity was enhanced as part of Corporal Expression Training, Storytelling and Pretend-play, and Improvisation and Role-play, without always specifying the role of each element. For this reason, even though we categorized different elements of DPT in order to clarify which specific elements impact creative processes, it seems equivocal to establish that only one element by itself is responsible for the enhancement of creativity.

The proposed synthesis and model are based in diverse literature, meaning that even if the cited literature showed positive effects of DPT on creativity, there are limitations. These limitations should be taken into account before citing any generalizations of the effectiveness of this model.

The biggest limitations are, in one part, a lack of well-designed protocols in certain studies that made it impossible to include many of them in the review. This is a common issue in drama-based literature, as most studies do not present complete reports, often missing critical analysis of results and deeper statistical analyses. This subsequently makes it difficult to predict the effects of specific elements of training beyond the authors’ interpretations, and replication possibilities are therefore limited.

Consequently, we propose to be very specific regarding the description of protocols, citing the duration of the intervention: “how many hours did the intervention last?,” “how many times a week did it occur?,” “for how long?” while also being specific about the activities carried on as part of the training: “how was the training constructed?,” “which type of activity was carried out first?,” “which one was carried out last?,” “did every session have the same structure?,” or “which elements of DPT were present during the intervention?” Furthermore, we propose to describe the variables that were measured and the instruments that were used to measure those variables. Within the literature reviewed, we had to dismiss interesting studies owing to the fact that the variables weren’t clear. For example, creativity or empathy development is referred to without defining the terms or the instruments used to measure them. It is also established that DPT had an impact on creativity, but without being specific on how creativity was measured. How Instruments were constructed or their validity is not fully explained, and results are reported vaguely without specifying effect sizes, correlations or even the significance of results. Consequently, more detailed descriptions of variables and results are also required.

In this line, the categorization of the elements of DPT helps us understand what are the specific creative outcomes that have been developed (or could be developed) through a particular element of DPT or through an ensemble DPT. Nevertheless, not all the studies reviewed offer a detailed description of the activities and elements of each session, making it difficult to establish whether one of the elements of DPT was not present in the training where creative outcomes were measured. Future research in the area should consider providing a more complete description of the approach, explicitly stating the final objective of the proposed training. A description of the different types of activities or elements used could also be important information that could be used to corroborate or update the proposed model, with the aim of contributing to the understanding of the process of creative thinking through DPT. With this kind of structure complementing by observations, impressions and theories of researchers and artists, we may develop more holistic, concrete and valid proofs to ensure the positive impact of DPT and its elements on children’s development.

Following this lead, even though this review presented evidence based on experimentation, some elements of DPT are related to perspectives that go beyond the scope of statistical findings. In other words, these kind of pedagogies and works based on dramatic art, demonstrate that scientific evidence could benefit from arts and humanities for complementing results, as happened with the case of Kharkhurin’s (2014) concept of creativity described for understanding creative outcomes through the corporal expression element of DPT. A more anthropological perspective of drama would explain creative thinking through symbolization and transformation abilities, by the transformation of social interrelations into symbolic representations of reality. In other words, social drama would impact creativity through a ritualistic metaphorical representation of social communities (Turner, 1975; Tydeman, 1988). For example, pretense is the metaphor of reality. Some studies have related metaphors to creativity related outcomes, as innovative thinking (Holstein, 1972) as well as metaphor, seen as the capacity of transforming reality by “existing” in both a fantasy and a real world (Bolton, 1979) is given thanks to DPT. In summary, sociological or anthropological perspectives of creativity and drama give us vast possibilities of discussion that could be used to complement quantitative analyses in future work.

On the other hand, The size of our sample seems insufficient for drawing further conclusions, as the majority of drama-based pedagogies’ literature corresponds to trainings focused on academic outcomes, like those included in the meta-analysis of Lee et al. (2015), and not DPT or creative drama trainings. Thus, we assume a possible involuntary exclusion of pertinent studies.

In any case, the vast majority of the studies showed that a drama-based pedagogy following the approach of DPT, develops creative thinking and creativity related processes in children. From our perspective, these findings have implications principally for school teachers and drama pedagogies, as well as for different professionals and actors in the educational and psycho-educational fields. We suggest further investigation of the elements of DPT that enhance creative thinking and a more thorough report of the studies carried out. Therefore, we believe that drama-pedagogy studies could benefit from an interdisciplinary approach in order to conduct studies that could gather the experience of artists and educators, but at the same time also report results and analysis of DPT practices in a more scientific way. This could benefit not only scientific research in the creativity field, but will also be beneficial for artists who want to understand the implications of dramatic art for the development of creativity, as well as for educators who are willing to develop the creative skills of children and adolescents in their classrooms.

## Author Contributions

M-PC wrote the manuscript and with support of MB and FZ. FZ coordinated and supervised the research review. All authors provided critical feedback and helped to shape the analyses and manuscript.

## Conflict of Interest Statement

The authors declare that the research was conducted in the absence of any commercial or financial relationships that could be construed as a potential conflict of interest.

## Footnotes

1 An integral and creative child should be understood as a child that has developed his/her socio-emotional competencies and creative thinking abilities.
